# Towards the implementation of home-based phantom limb pain training facilitated by a textile-electrode system: lessons learned from a pilot study

**DOI:** 10.1186/s12984-026-01923-w

**Published:** 2026-03-11

**Authors:** Li Guo, Anna Björkquist, Maria Munoz-Novoa, Morten B. Kristoffersen, Max J. Ortiz-Catalan, Leif Sandsjö

**Affiliations:** 1 Swedish School of Textiles, University of Borås, Borås, Sweden; 2 Department of Clinical Neuroscience, Institute of Neuroscience and Physiology, Sahlgrenska Academy, University of Gothenburg, Gothenburg, Sweden; 3 Chalmers Industriteknik, Gothenburg, Sweden; 4 Department of Engineering Technology, Technical University of Denmark, Ballerup, Denmark; 5 Prometei Pain Rehabilitation Center, Vinnytsia, Ukraine; 6 Center for Complex Endoprosthetics, Osseointegration, and Bionics, Kyiv, Ukraine; 7 Faculty of Caring Science, Work Life and Social Welfare, Department of Work Life and Social Welfare, University of Borås, Borås, Sweden; 8 Department of Industrial and Material Science/Div. Design & Human Factors, Chalmers University of Technology, Gothenburg, Sweden

**Keywords:** Phantom limb pain, Phantom motor execution, Textile electrodes, Home-based rehabilitation, Pilot study, User experience

## Abstract

**Background:**

Phantom limb pain (PLP) affects many individuals with limb loss and often requires long-term treatment. Phantom motor execution (PME), which involves decoding phantom movements through myoelectric pattern recognition and real-time feedback, often delivered via extended reality (XR), has shown promise in reducing PLP. However, its use in home settings is limited by usability and the cost of traditional disposable (Ag/AgCl) electrodes. Textile-electrode systems may offer a more user-friendly and sustainable alternative, improving the practicality of home-based PLP training.

**Objective:**

This pilot study aimed to explore the potential of using a textile-electrode system for self-administered, home-based PME interventions, focusing on user perceptions and experiences through qualitative data.

**Methods:**

Five participants with lower and one with upper limb amputations were provided with a textile-electrode system (*Textrode-band*, a study-specific prototype developed at the University of Borås) connected to a PME system (Neuromotus, Integrum AB, Sweden). Participants used the system at home for 24 weeks: 12 weeks of guided training (video sessions and mobile reminders), followed by 12 weeks of unsupervised training with only a weekly emailed training plan. The primary outcome was user acceptance and usability of the system, assessed through semi-structured interviews. Secondary outcomes included training frequency and continuity over two intervention periods. Self-reported PLP and quality of life data were collected to support interpretation but are not reported here.

**Results:**

Participants generally found the textile-electrode system comfortable, easy to use, and feasible for independent use at home. Two participants reported reductions in PLP intensity or frequency, and one described improved sleep quality and several reported that the system allowed them to integrate the training into daily routines, improving their sense of control and self-management. However, some challenges with motivation and consistency, especially during the unsupervised phase, indicating the need for ongoing support.

**Conclusion:**

The textile-electrode system shows potential to support self-administered, home-based PME offering a user-friendly and practical alternative to traditional electrodes. Future research should focus on improving system robustness, integrating engaging training strategies, and developing support frameworks that balance user independence with necessary guidance. The findings also provide insights into advancing home-based rehabilitation for other neuromuscular conditions requiring long-term rehabilitation.

**Supplementary Information:**

The online version contains supplementary material available at 10.1186/s12984-026-01923-w.

## Background

Phantom limb pain (PLP) is a condition in which individuals experience pain in a limb that has been amputated [1]. It is a chronic condition that affects a large proportion of amputees, with reported incidences varying between 42% and 85% among individuals who have undergone limb amputation [2–4]. PLP can be experienced with different intensities and sensations, including sharp, burning, or aching sensations [5]. Despite being recognized for centuries, PLP remains poorly understood, and its persistence poses a challenge for both clinicians and patients [6, 7]. In addition to direct pain, PLP often leads to long-term psychological distress, mobility issues, and a significant reduction in quality of life, including limitations in sleep and participation in daily activities [8].

Traditional pain management methods such as medications often fail to provide sufficient PLP relief [9, 10], which has led to alternative treatments being explored, such as mirror therapy, transcutaneous electrical nerve stimulation (TENS), acupuncture, and, more recently, phantom motor execution (PME). PME is a therapeutic approach that decodes myoelectric signals from the muscles of the residual limb to infer the intended movement of the phantom limb [11]. The myoelectric signals are interpreted via a pattern recognition system, allowing patients to control a virtual limb in real-time, typically visualised through extended reality (XR), including (non-immersive) virtual reality (VR), augmented reality (AR) and serious games/gaming [12]. The combination of PME with XR creates an interactive and immersive training experience that is directly driven by the user’s own motor intent [7]. This approach not only strengthens residual muscle activation but also facilitates purposeful engagement of both central and peripheral motor structures, which may promote adaptive neuroplasticity and help disentangle pain processing circuits [7]. Previous PME studies have shown promising results in reducing PLP by following a training program of 15 sessions, which are conducted 2–3 times per week for approximately 1.5 h each [13]. To enhance the long-term effect, high-dose and repetitive training is needed. This has led to a growing interest in making PME more accessible through self-administered, home-based systems [14]. However, certain challenges remain, particularly related to the self-administered placement of the electrodes used to capture myoelectric signals [15, 16]. The most common type of electrode used in PME interventions is disposable (Ag/AgCl) electrodes, which are placed on the skin to capture myoelectric signals. While effective, these electrodes present several drawbacks: they rely on adhesive materials that can cause skin irritation, their placement must be precise for accurate signal acquisition, and their single-use nature increases operational costs [17, 18]. Additionally, they can be challenging to place, especially for individuals with only one able hand. For many users, these issues hinder compliance in home-based interventions, highlighting the need for more user-friendly, self-administered, and sustainable alternatives.

To address these limitations, several alternative electrode technologies have been developed. These include reusable dry metal electrodes [19, 20], conductive polymer-based electrodes [21, 22], and capacitive electrodes [23, 24]. While these approaches can avoid adhesive-related irritation and reduce waste, they present trade-offs, such as rigidity, the need for precise skin contact, or limited breathability, which can compromise comfort and usability and may limit long-term wearability.

Textile-integrated electrodes have emerged as a promising solution in this context [15]. They offer several advantages over disposable electrodes, such as the integration of sensing functions into soft, flexible, and breathable textiles and the ability to minimize skin irritation. Their reusability also makes them a more cost-effective and sustainable solution for repeated use. Furthermore, textile electrodes can be integrated into everyday garments, making them more convenient for users to wear and thus potentially improving adherence to home-based training [25]. Preliminary studies suggest that textile electrodes may be used as viable alternatives to disposable (Ag/AgCl) electrodes in PME interventions for PLP [15]. Building on these initial findings, specifically in the reported pain reduction benefits of PME and addressing limitations of conventional electrodes, this pilot study explored the combination of PME with a textile-electrode system for self-administered, home-based PLP management. It focuses on the usability and user experiences of the textile-electrode system, as perceived and reported by participants, along with their overall perspectives on integrating such a system into daily life. Additionally, this study provides insights into the methodological and practical challenges of conducting research in participants’ home environments, contributing valuable lessons to guide the design of future feasibility and clinical studies. This pilot did not assess safety or effectiveness outcomes.

## Methods

### Study design

This pilot study was designed as a single-arm, exploratory the use of a textile-electrode system for self-administered, home-based PME training in individuals with PLP. Rather than evaluating safety or treatment effectiveness, the study focused on understanding participants’ experiences with the textile-based solution in a real-world home setting.

The primary outcome was the usability and acceptability of the textile-electrode system, as perceived by users. This was assessed through qualitative data collected via semi-structured interviews. The interviews explored users’ perspectives on system functionality, comfort, ease of use, and overall satisfaction. The secondary outcome focused on participants’ engagement with the PME training over time, as captured by training frequency, duration and continuity when external support was reduced. These data were obtained from system usage logs and participant interviews. In addition, we collected self-reported changes in PLP intensity and perceived impact on quality of life using questionnaires. As this was an exploratory study with a small sample size, the quantitative data were intended to complement the qualitative findings to illustrate individual trends, rather than to draw general conclusions about effectiveness.

## Intervention

**Fig. 1:**
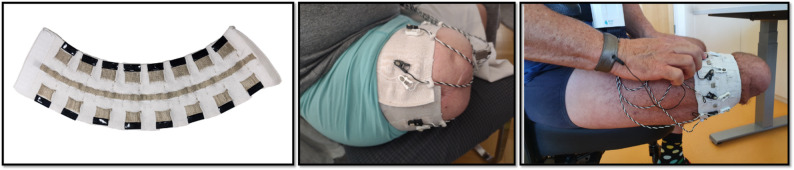
Textrode-band (left) and participants wearing the Textrode-band (middle and right): the middle panel demonstrates application on a shorter residual limb, whereas the right panel illustrates usage on a longer residual limb

The intervention used Neuromotus™ (Integrum AB, Mölndal, Sweden), an sEMG acquisition and analysis system, in combination with a “*Textrode-band*”, i.e., the textile electrode system (Fig. 1 left). The *Textrode-band* is a *textile-band* that consists of eight pairs of knitted electrodes in a bipolar configuration and one long central electrode, enabling its use as a monopolar electrode when needed. The curved design allows the band to conform to the cone-like shape of most amputated limbs for an optimal fit. The materials and construction of the Textrode-band were described in a previous study [15], which also demonstrated favourable signal quality and signal-to-noise ratios (SNRs) for deciding intended limb movements supporting their feasibility and reliability for use in PME training.

The PME training, supported by VR/AR, has been well described in previous studies for both the upper [26] and lower limbs [27]. Briefly, myoelectric signals from the residual limb are decoded and mapped to control a virtual limb in real time. The virtual limb is displayed on a conventional monitor in either VR or AR. VR enables the use of virtual environments, including games and task-based training. In AR, the virtual limb is projected over the user’s residual limb via webcam, creating a visual illusion of the missing limb moving within the participant’s environment. Together, these technologies aim to increase user engagement and promote sensorimotor integration and cortical reorganization, which are considered key for treatment adherence and mechanisms in reducing phantom limb pain [26].

Each training session followed a structured sequence: (1) a self-assessment of pain, (2) placement of the electrodes, in this study, the Textrode-band, (3) real-time verification of myoelectric signals, (4) recording of myoelectric signals from the residual limb muscles through a series of standard/prescribed limb movements (e.g., knee flexion/extension) to train the system in identifying motor intentions, and (5) practice of motor execution in: VR or AR 6a) gaming: controlling a virtual racing car or break-out bar from intended phantom limb movements or 6b) matching random target postures of a virtual limb in VR.

The training includes several levels of difficulty and was assigned depending on each participant’s ability to control the system. The participants typically started with two movements involving a single degree of freedom - DoF (e.g., wrist flexion/extension or hand open/close) and could progress to multiple single DoFs at the time or to more complex movements requiring two DoFs (e.g., simultaneous hand open/close and wrist flexion/extension). Progression to a higher level followed when participants successfully completed tasks at the current level, making the exercise gradually more challenging over time. Conversely, if participants struggled to meet the requirements, the difficulty was reduced.

**Fig. 2:**
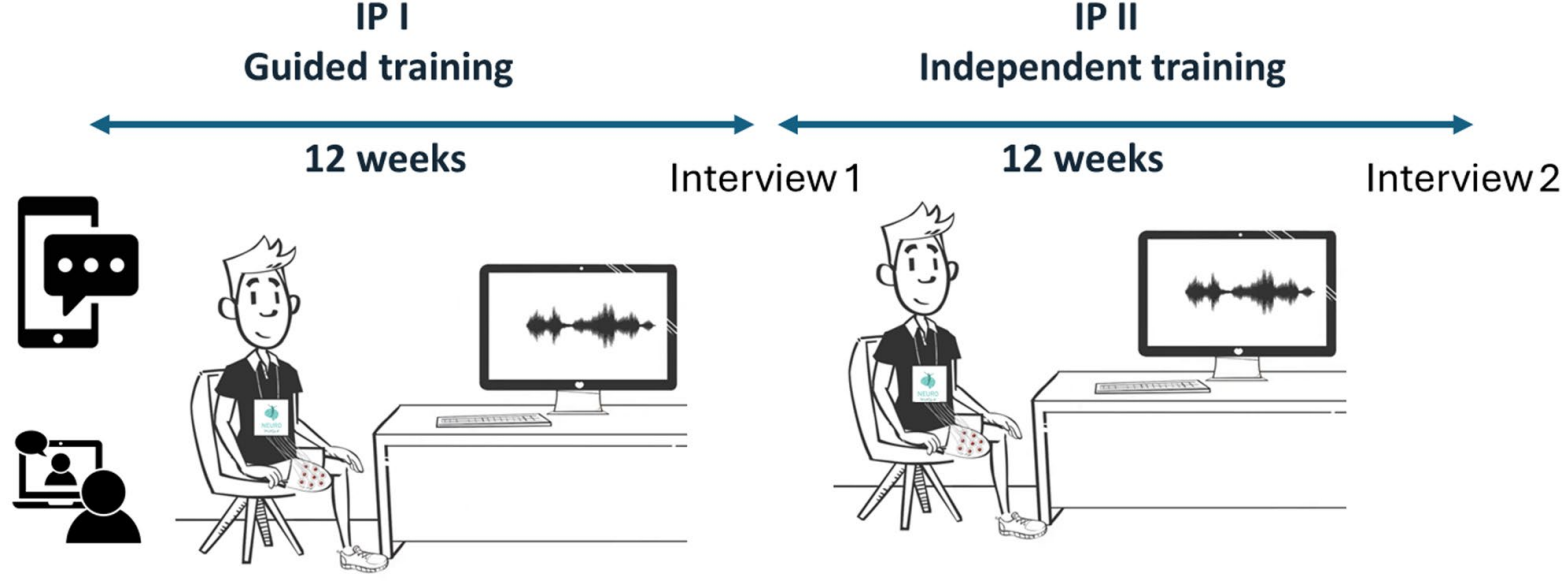
Intervention plan. IP I had support at hand by video conference whenever the participant wanted, and the training sessions were also prompted by text messages. IP II had no online support or reminders, but a weekly training plan was e-mailed each Monday

In total, the intervention spanned 24 weeks and consisted of two intervention periods (Fig. 2). Intervention Period I (IP I) lasted 12 weeks, during which the participants were instructed to train every other day. On each scheduled training day, a mobile text message reminder was sent, and professional guidance (provided by A.B., L.G. or M.M.N.) was available via video call. At the end of the IP I, the first round of interviews was carried out.

Intervention Period II (IP II) spanned another 12 weeks, during which participants continued the same exercises in the same manner as in IP I but without SMS reminders or personal guidance from the research team. The participants, however, received tailored training recommendations each Monday via e-mail. This reduced level of support was introduced to assess how participants maintained training continuity and were able to incorporate PME into their routines independently. At the end of the second period, the second round of interviews was conducted, and the final data were gathered (A.B).

### Screening visit and pre-intervention

Potential participants were identified by healthcare professionals or recruited among individuals who had previously been in contact with the research group. Individuals with sufficient residual limb length and detectable sEMG signals in the residual limb were eligible if they experienced chronic PLP and had not undergone PLP treatments or reported related pain reductions in the three months prior to screening. Acute PLP cases, unstable prosthetic use, or insufficient sEMG signals, as assessed by the researcher, were grounds for exclusion. Prior to engaging in any study-related activities, all potential participants provided informed consent. The study was approved by the Swedish Ethical Review Authority (Dnr 2021–03272). All participants were recruited, provided informed consent, and received initial training in Sweden. Home-based training and remote data collection (questionnaires and usage logs) were conducted via the study system in accordance with the approved protocol.

During the screening visit, participants who fulfilled the inclusion criteria and chose to participate were introduced to the PME training system. Each participant was measured to select the most appropriate Textrode-band size, according to length, circumference, and shape, ensuring a good fit to the residual limb (Fig. 1) on the left shows usage on a longer residual limb. Each participant was then instructed on how to use the PME training system. This ensured that all the participants were familiar with the system before starting to use PME training at home.

Following the screening visit and typically two weeks before the intervention started, participants attended a pre-intervention session where they practiced using both the Neuromotus™ device and the Textrode-band at the research lab with supervision. While the goal was to prepare participants for independent operation of the system, support from the research team was available by phone/Zoom throughout the home period to assist with any issues or questions. This ensured that participants could manage the system at home while still having access to professional guidance if needed. Once the two-weeks pre-intervention training period was complete, the participants were provided with two Textrode-bands and a Neuromotus™ device to use in their homes.

## Participants

Six participants with varying levels of amputation, prosthetic use and PLP management strategies were included.

Key characteristics such as age, prosthetic use, PLP characteristics, and previous PME experience are summarized in Table 1.

## Data collection

To assess the primary outcome, i.e., user acceptance and usability of the textile-electrode system, semi-structured interviews were conducted at the end of each 12-week intervention period (IP I and IP II) (Fig. 2). The interviews aimed to collect in-depth feedback on participants’ experiences using the PME system at home and how it affected their daily routines and overall quality of life. The semi-structured interview guide included four topics, i.e., (1) experiences of previous treatment using Ag/AgCl electrodes (if any); (2) experiences of the current intervention; (3) experiences from using the Textrode-band and PME system; and (4) any changes in health status and behavior the participants believed could be related to the PME training (interview guide – supplementary A).

Secondary outcomes focused on participants’ engagement over time and continuity of use across both intervention periods. System usage, particularly training frequency and session duration were collected from the usage logs.

In addition, self-reported PLP intensity and health-related quality of life were collected using the Questionnaire for Phantom Limb Pain (Q-PLP) and EuroQoL-5D-5 L (EQ-5D-5 L) [28] at the end of each training session. The Q-PLP is a 16-item questionnaire based on the short form of the McGill Pain Questionnaire [29] and study-specific questions used in previous studies [27, 30] addressing multiple aspects of the PLP, including intensity, character, duration, frequency of pain and weighted pain distribution.

### Data analysis

All interviews were transcribed verbatim for qualitative analysis. Given the small number of participants, a deductive content analysis approach [31] was applied, framed by four key categories, i.e., *Outcome*,* Motivation*,* Usability*, and *Support*,* which were* previously established in a similar home-based intervention study [32], and how these categories might have affected interest *in continuing to use* the system under evaluation [32]. A thorough review of the transcripts was conducted (L.G. and A.B.) to gain familiarity with the data and develop an in-depth understanding of the participants’ experiences. The transcripts were then coded according to the four framing categories, with key excerpts assigned to the relevant themes. To ensure consistency and rigor, three researchers (L.G., A.B., and L.S.) compared the initial coding and worked collaboratively to refine the categorizations, resolving any discrepancies through discussion. Sub-themes within each category were identified by referring to an established framework [33], which provided additional structure and deeper insight into the data. The team revisited transcript excerpts during the validation process to confirm alignment with the chosen categories and to ensure comprehensive representation of the data. Finally, the categories and sub-categories were analysed in relation to the study objectives, with a focus on the use of the textile-electrode system for home-based PLP training.

Statistical analyses of the quantitative data (e.g. Q-PLP and EQ-5D-5 L) were not conducted, as the number of participants was too low to study how the intervention may have affected PLP outcomes.

**Table 1:** Participant characteristics

Participant	Basic information		PLP characteristics	Previous treatments	Use of prosthesis
S1	**Age range**	40–50 years	Moderate, Constant, Increased pain at night and during inactivity.	Painkillers, No PME experience	-Bone-anchored/osseointegrated prosthesis, -Use it on a daily basis. -Anticipated a newer prosthesis during the study.
	**Sex**	Male			
	**Time since Amputation**	> 25 years			
	**Cause of Amputation**	Trauma			
	**Level of Amputation**	Above Knee			
S2	**Age range**	60–70 years	Severe, Constant, Severe daily episodes, Linked to mood and weather.	Painkillers, spinal TENS, mirror therapy, No PME experience	-Bone-anchored/osseointegrated prosthesis -Use it frequently
	**Sex**	Male			
	**Time since Amputation**	> 30 years			
	**Cause of Amputation**	Trauma			
	**Level of Amputation**	Above Knee			
S3	**Age range**	50–60 years	Moderate, Constant, Severe at night	Painkillers, mirror therapy, Has PME experience	-Bone-anchored/osseointegrated prosthesis -Use it on a daily basis
	**Sex**	Male			
	**Time since Amputation**	~ 10 years			
	**Cause of Amputation**	Trauma			
	**Level of Amputation**	Above Knee			
S4	**Age range**	50–60 years	Moderate, A few times a week, Increased pain at night	Painkillers, acupuncture, mirror therapy, Has PME experience	-Socket prosthesis -Use it on a daily basis
	**Sex**	Male			
	**Time since Amputation**	~ 20 years			
	**Cause of Amputation**	Trauma			
	**Level of Amputation**	Above Knee			
S5	**Age range**	40–50 years	Mild to Moderate, A few times a week, Pain episodes vary with activity	Mirror therapy, No PME experience	Not using any prosthesis
	**Sex**	Female			
	**Time since Amputation**	~ 5 years			
	**Cause of Amputation**	Trauma			
	**Level of Amputation**	Above Elbow			
S6	**Age range**	60–70 years	Mild, Occasional episodes, Manageable with painkillers	Massage therapy, occasional painkillers, No PME experience	-Bone-anchored/osseointegrated prosthesis -Use it frequently
	**Sex**	Male			
	**Time since Amputation**	~ 8 years			
	**Cause of Amputation**	Trauma			
	**Level of Amputation**	Above Knee			

## Results

The results are organised according to the study’s outcomes, beginning with participants’ experiences of training at home. This is followed by an overview of participants’ participation across the two intervention periods, including training frequency and duration, and dropout or retention.

### Interview results

The qualitative data were analysed using four pre-defined categories, i.e., Outcome, Motivation, Usability, and Support [25] (detailed categorization - Table 2 appendix). These categories were used to organise and interpret participants’ feedback about their experiences and engagement with the system and challenges navigated during the intervention.

### Outcome

The “outcome” category, related to the participants’ perceptions of immediate gain from taking part in the PLP intervention, revealed some improvements in pain management, decreased medication intake and enhanced quality of life. Some participants reported that PME training at home led to noticeable reductions in PLP occurrence and intensity. One participant mentioned, *“When I practiced every week*,* I didn’t feel the pain as frequently*,* nor did I feel the pain as severe. However*,* when I did not practice for three weeks (note: due to vacation)*,* I had pain every day”* (S5). Another noted a major reduction in pain *“from absolutely excruciating*,* screaming pain to more of a groaning*” (S4). Furthermore, some participants gained more control over their nonpainful phantom sensations. S3 mentioned that with continued practice, he was able to ease the sensations in his phantom toes, which he believed to be a positive outcome of the training.

A decrease in pain medication use was another positive outcome reported by some. One participant (S4) reported that pain relief from the training reduced the need for painkillers and the side effects from using them: *“I’ve reduced medicine intake*,* which has reduced my toxin intake*,* and I can absolutely feel the difference in that”* (S4). This reduction in medication also contributed to a greater sense of calm and improved overall well-being. *“There’s a calmness that has entered my system”* (S4). However, not all participants experienced pain reduction. As mentioned in the previous section, two participants reported increased pain, which ultimately led them to discontinue their participation. However, we cannot ascertain whether this pain was associated with the PME treatment.

In terms of quality of life, S4 and S5 expressed themselves felt more empowered and in control of their lives. One participant shared, *“It just gave me a bit more power; I was less pressurized [stressful]. Physically*,* it [I] was just faster and easier*,* much better”* (S4). This sense of independence extended beyond physical well-being, as participants also reported improvements in mental health, with one stating, *“There’s been a mental improvement*,* a psychological improvement*,* a toxicity improvement [reduction]. It’s quite astonishing to notice the difference”* (S4).

### Motivation

We explored participants’ motivation for both joining the project and continuing to use the system at home after the project concluded. The primary motivation for joining was the potential for PLP reduction. Beyond pain relief, two participants expressed a strong desire to contribute to research by participating in research studies, seeing their involvement as a way to help others who might benefit from future advances: *“I’m happy to join and continue to help*,* even if I do not have a problem*,* it can help others*,* that is good*,* all for the sake of research”* (S6). The participants were also motivated by the opportunity to learn and engage with new technology. One participant described the experience as *“fantastic*,*”* stating, *“I think it is kind of cool that you can measure muscle activity in this way; it’s feedback that motivates you to train”* (S6). This fascination with innovative methods increased their commitment to the project.

The motivation to continue the PME training at home after the project was driven not only by the ongoing benefits of pain management but also by a sense of hope brought about by technology itself. The participants viewed the new technology as a tool for sustained progress and improvements in quality of life. One participant, who returned to work after training, mentioned that his fear of sudden pain was reduced, giving him hope. He shared that *“now I can sleep*,* and I can dream”* (S4), which he associated with a renewed sense of hope and relief in his life.

### Usability

The participants highlighted the simplicity and comfort of the system as key factors facilitating training at home. All participants felt that the system was easy to learn, and they gained confidence in using it independently over time. The design was described as intelligent yet straightforward, with some participants initially noting minor placement challenges that were resolved through practice. One participant stated, *“It became quick and easy*,* you get used to it [preparing the Textrode-band]. I’m almost an expert in that now”* (S4). Another participant shared how they initially needed help but eventually managed independently: *“For me*,* I just asked my family to help me fix it for the first few times. Later*,* it was me”* (S5).

The Textrode-band was praised for ease of use and comfort, particularly by experienced users who were familiar with alternative solutions. One participant noted, *“It’s a thousand times better [than single use electrodes] and I do enjoy not having my hair pulled out of my leg every time I remove them [Textrode-band]”* (S4). They also mentioned that the Textrode-band significantly reduced the preparation time needed before training compared with Ag/AgCl electrodes, as it eliminated the need for precise placement and keeping track of electrode positions.

In terms of system robustness, reliability and overall accuracy, most participants appreciated that the Neuromotus (both software and hardware) and the Textrode-band performed reliably during training sessions. *“Technically*,* the system works brilliantly; nothing looks like it’s wearing out yet. It seems to be in very good condition; it’s very good quality”* (S4). One participant highlighted the reliability of the Textrode-band, stating, *“It [the Textrode-band] finds the signals*,* that’s guaranteed*,* and you did not have to turn it*,* you just put it on”* (S3). Another participant, despite initial concerns about the durability of the Textrode-band, stated, *“I thought this [the Textrode-band] will break; it will fall apart [fall off the limb]*,* but it attached great with Velcro”* (S6). The software was also praised for its consistency, with one participant sharing: *“The program [software] has worked great; there has never been a problem starting it”* (S6). However, despite the general positive feedback, some concerns about accuracy were reported, particularly when participants trained with the *break-out bar* game. This led to physical and mental fatigue for one participant (S2), which may have contributed to the decision to leave the study.

Participants also appreciated the system’s availability and compatibility with their daily routines. The possibility of using the system at home according to their own schedule made it feasible to integrate PLP training into everyday life, and participants could adapt their use of the system to their schedules and personal needs. One participant, appreciating the freedom to decide when to perform PME training, stated, *“I am truly busy from Monday to Sunday without any rest. The only rest is that when my children fall asleep*,* I can sit down and take a bath at night*,* and then I can practice my hands [with the Textrode-band system].”* (S5). Another participant created a routine that fit their schedule, explaining, *“I made it part of my weekly routine*,* like people going to church on Sundays. I dedicated that time to myself—for my mind and body—taking the weekend to focus without feeling rushed.”* (S4).

Finally, the system’s design saves time and effort compared with traditional methods. One participant explained, *“I told my facilitator*,* if I should do this [training] at home*,* you have to find the position of the electrodes and I have to draw with a marker and then make a tattoo where they should be*,* so it remains*,* but then the band arrived*,* and it works great”* (S3).

### Support

The participants valued the support they received from the research team throughout the study. This included personal guidance, reminders, and a structured training plan. One participant emphasized the importance of receiving new challenges from professionals to avoid sticking to simpler exercises (S6). Many appreciated regular communication, with one noting, *“The weekly emails were clear and comprehensive*,* providing very good guidance” (S5).* The availability of timely support and strong connections with the research team was also highlighted as motivating factors for engagement (S4).

Support from family was provided to some participants during the study. For S2, his partner, who also serves as his personal assistant, assisted with the study and interviews. Owing to physical limitations such as difficulty in balancing on one leg, S2 needed assistance from his partner to apply the Textrode-band. Similarly, S5, the only upper limb amputee in the study, initially found it difficult to wrap the Textrode-band around her arm with one hand. Her daughter assisted with placing the band during the first few weeks. To address this issue, the band design was improved after three weeks by replacing the temporary Velcro closure with a permanent sewn design, allowing the band to be used as a tube. In practice, S5 was able to manage the band independently.

In addition to professional and family support, participants expressed a desire for a supporting community with others in similar situations. One participant noted that connecting with other people with limb loss would provide emotional support and reduce the isolation commonly felt by people with limb loss (S4). They also emphasized the emotional and psychological benefits of belonging to a support group, describing the research team as a *“support family”* that made a significant difference. *“I felt quite empowered like I have something I can do myself. I’m not relying on my facilitator or somebody else to do it for me; that was a big step”* (S4).

### Participant participation across intervention periods

Participation appeared to be a significant challenge; by the end of the study, three out of the six participants had discontinued due to different reasons.

**Fig. 3:**
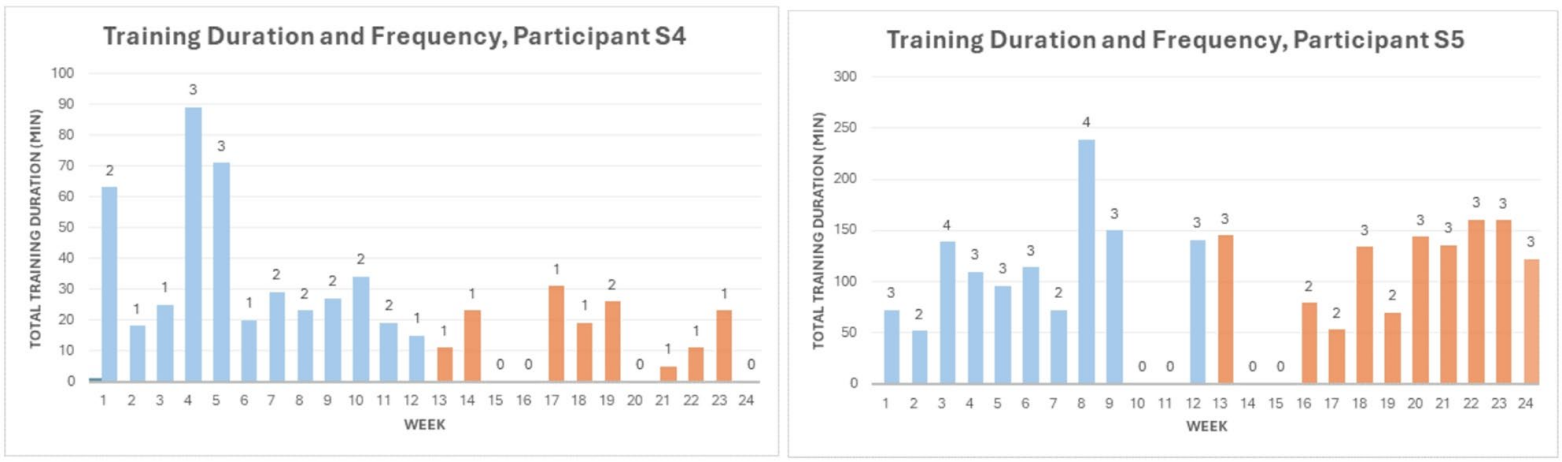
The participation patterns of S4 and S5 during IP I (blue) and IP II (orange): the bars represent the total weekly training duration, and the number on top of each bar represents the number of training sessions the participant completed in the corresponding week

Participants S4 and S5 completed both IPs, allowing for a comparison between the two periods. During IP I, S4 completed a total of 26 sessions, averaging approximately 2.2 sessions per week, with an average training duration of 22 min per session (Fig. 3, left). Despite some skipped sessions, S4 maintained a relatively consistent training schedule in line with the study’s recommendations for IP I. During IP II, without SMS reminders or direct professional guidance, S4 completed a total of 10 sessions. The average training duration per session for S4 dropped from 22 to 16 minutes between the IP I and IP II (Fig. 3, left). S5 maintained a relatively consistent training schedule, completing 30 sessions (2.5 sessions/week) in line with the study’s recommendations for IP I (Fig. 3, right), whereas 27 sessions were completed during IP II, with the average training duration increasing from 35 to 45 min per session (Fig. 3, right). Notably, S5 missed two training weeks (weeks 10 and 11) during her summer vacation travel and was unable to train in weeks 14 and 15 due to illness.

Participant S6 was only active in IP I but did not withdraw from the study. During IP I, S6 completed 11 training sessions, with an average training duration of 30 min per session. His inactivity during IP II was primarily due to his very low frequency of PLP episodes, which he reported occurring approximately once per month at the screening visit. Given that there was a minimal need for PME training, especially in the absence of structured requirements during IP II, S6 chose not to perform any exercises. Nevertheless, he remained engaged with the study and provided valuable feedback during post-intervention interviews conducted after both intervention periods.

S1 withdrew from the study after 9 weeks (IP I) due to unexpected pain caused by new prosthesis, an issue unrelated to the current study. S2 completed 13 sessions (IP I) over a span of 12 weeks before withdrawing because of increased pain associated with PME training. Additionally, S2 experienced technical issues with the device, specifically related to battery performance. He had to pause his training to exchange the device, which disrupted his routine and may have affected his motivation to continue. In the case of S3, pain became unbearable, significantly reducing his motivation to continue training after only 2 sessions (IP I). S3 did not explicitly attribute the increase in pain to the PME training itself.

## Discussion

The results of our study support the use of the textile-electrode system as a promising alternative to Ag/AgCl electrodes for home-based PLP training. All participants highlighted the comfort and ease of use of the Textrode-band, and those who had previously tried PME found it easier to perform routinely than when Ag/AgCl electrodes were used. By reducing setup time and allowing for flexible use at home, the Textrode-band was shown to facilitate sustained engagement, which is a critical factor in achieving long-term positive training outcomes.

Some participants in our study reported improvements in PLP management and quality of life, resulting in a reduction in pain occurrence and intensity. This confirms the utility of PME as a viable approach to PLP management when consistent engagement is crucial for its effectiveness and for maintaining positive outcomes [13]. A key insight here is that personalized, training schedules and flexible training regimens are fundamental to achieving optimal results, as participants benefit from autonomy to fit the interventions into their personal routines. The easy application of the Textrode-band contributes to this flexibility, as participants can use it independently, allowing them to feel more in control of their training program. Among the two participants who trained during both intervention periods, we observed continued use of the system, with only minor adjustments to the training frequency. One participant slightly increased their training time, whereas the other reduced their training time during the second period. This consistency suggests that the positive outcomes achieved through PME training, combined with the usability and comfort of the Textrode-band, may support long-term engagement. These participants demonstrated that the ability to adapt the intervention to their routines, guided by the working schedules they established, was key to maintaining regular use and managing PLP. However, the decrease in training frequency observed during IP II, when reminders and professional guidance were discontinued, raises the question of whether it is important to incorporate additional, continuous support options, such as automated reminders, to sustain engagement in home-based interventions over extended periods or whether this was the result of an individual adaptation of training frequency according to actual needs to control the PLP. The study also suggests the potential benefits of incorporating community-based peer support into home-based PLP interventions. Future studies should explore individual choices regarding the occurrence of PME training when it is offered at any time (free use) and the potential value of a community network where participants can share strategies, progress, and support. This could complement professional guidance and help improve long-term engagement and outcomes.

Owing to each session’s identification of myoelectric patterns from test contractions, the integrated textile electrodes successfully captured myoelectric signals suitable for pattern recognition and movement discrimination. However, we found that the sensitive/fine-tuned control needed during the game-based exercises was challenging for some participants. While the exact cause remains uncertain, it is possible that this difficulty is related to the quality of signals captured by the Textrode-band. Further research is needed to explore whether signal quality issues, such as variations in SNR, might contribute to these challenges and how potential improvements in signal processing or electrode design could address them.

Finally, our findings highlight the importance of more intuitive and engaging training platforms to keep users motivated by improving the user experience. Features such as gamification and personalized feedback may help increase long-term commitment. Further work should address not only the technical challenges but also focus to improving the emotional and psychological dimensions of the training experience [34].

### Lessons learned

This study reveals several key areas for improvement. We observed that reported questionnaire values sometimes did not align with the qualitative interviews. For example, one participant consistently rated PLP as 0–1 on questionnaires yet described in interviews a significant reduction in overall pain intensity. This mismatch occurred because the participant interpreted the self-reports as assessments of momentary pain during training rather than as reflections of their average pain experience. As a result, the quantitative data underestimated perceived benefits. A key lesson is that future home-based studies should employ pain assessment strategies that more clearly capture fluctuations and average pain levels, for example through diaries, repeated daily entries, or ecological momentary assessment methods [35].

Second, both severe pain and too little pain contributed to the discontinuation of the interventions fully or partially, which make us reflect, in future research, on the need to investigate prognostic factors that can help define the target population in terms of inclusion and exclusion criteria. The results of this study indicate that PLP intensity and occurrence should be considered. Another important consideration during participant recruitment is the ability to apply the Textrode-band independently, as participants in this study with mobility limitations, short residual limbs, and sometimes reduced dexterity experienced some struggle with the setup, which might have affected their participation. Assessing these variables should guide the development of criteria for future studies to ensure participant suitability.

Third, we noted that while information about potential temporary increases in PLP was communicated both verbally at the screening visit and in the informed consent, one participant still reported an initial increase in pain that led him to exit the study. This suggests that for future home-based studies, where continued communication can be challenging, researchers may need to employ repeated reinforcement of essential study details, particularly concerning potential side effects or discomfort. Written summaries, follow-up calls, and even additional documentation highlighting potential challenges could improve participants’ understanding and readiness to use or adapt the intervention to their personal needs.

Finally, we also reflect on the long-term implementation of home-based PME interventions for PLP and other rehabilitation contexts. While the independence they can offer benefits such as convenience and flexibility, their success also depends on having strong support systems in place. A hybrid approach that combines autonomy with structured support, such as remote professional guidance, peer support networks, and automated reminders, could enhance adherence and engagement in home-based PMEs. Such a hybrid approach may be essential for translating the benefits of PME from controlled studies to real-world applications.

### Limitations

This study has several limitations that should be considered when interpreting the results. The primary limitations are the low number of participants and the limited number who completed the full protocol. The small sample size restricts the diversity of experiences and outcomes captured, thereby reducing the ability to gain a comprehensive understanding of the effectiveness and challenges associated with home-based interventions. A larger and more varied participant group would provide richer insights and enhance the generalizability of the findings.

Moreover, a significant portion of the data and insights derived from this study predominantly come from two participants. This reliance on detailed accounts from only two participants, while valuable for in-depth qualitative analysis, may skew perception of the intervention and the overall participant experience. It limits the scope to individual experiences, which might not fully represent the broader user base.

## Conclusions

To explore user perceptions and experiences, this study evaluated the use of a textile electrode system Textrode-band for self-administered, home-based PME interventions aimed at reducing PLP. The results demonstrate the system’s potential, offering benefits such as usability, reduced setup time, and facilitation of individual integration into daily routines. However, challenges remain, including the need for engaging training options, improved system performance, and enhanced support procedures. Future development should prioritize improving system robustness and integrating serious gaming training platforms to support gamified interventions. In addition to technology, effective PME implementation may require a broader approach that includes adaptable training plans, professional guidance, and peer support. Future research should explore how these elements contribute to user engagement and long-term adherence. These insights contribute to the advancement of home-based rehabilitation not only for PLP but also for other neuromuscular conditions where self-administered, technology-assisted interventions could play a significant role in improving rehabilitation outcomes.

## Supplementary Information

Below is the link to the electronic supplementary material.

Supplementary Material 1

## Data Availability

The data supporting the conclusions of this study are available upon reasonable request from the corresponding author. Owing to GDPR, data sharing is limited to protect participant confidentiality.

## Appendix 1

See Table 2 here.

**Table 2:** Categorization of key findings in relation to the chosen framework

Framework Category	Our findings	Description/meaning unit	Excerpts
**Outcome**	**Pain management**	Interventions led to pain related outcomes, such as, pain relief, changes in the types of pain experienced and the awareness of the phantom pain/sensation.	*“When I practiced every week before*,* I didn’t feel the pain as frequently as before*,* nor did I feel the pain as severe. But when I didn’t practice for three weeks (note: vacation)*,* I had pain every day.”-S5* *“On average I’s say that the intensity has reduced about sixty to seventy% from absolutely excruciating*,* screaming pain to more of a groaning” -S4* *“I start to feel that I feel the toes respond to it then… if I do it for a while*,* I can get it to lighten up a little. That’s the positive thing that I learned it. It wasn’t something I was doing before.” -S3*
	**Quality of life**	Added value to the users about their conditions, treatment options and the everyday life.	*“Being more aware of the situation… This is a very significant thing for me*,* it turns out my values and what I would like to treat my body like*,* it’s massive.” -S4* *“(to do it at home) It just gave me a bit more power*,* I was less pressurized actually. Physically it was just faster and easier*,* much better.” -S4* *“I’m generally feeling a bit fitter because I got more mobile time. That’s a very distinct difference.” -S4* *“There’s been a mental improvement*,* a psychical improvement*,* a toxicity improvement*,* psychological improvement… it’s quite astonishing to notice the difference.” -S4*
	**Medication reduction**	A decrease in reliance on medications due to pain management and/or the change of life quality.	*“I’ve reduced medicine intake which has reduced my toxin intake*,* and I can absolutely feel the difference in that.” – S4*
**Motivation**	***To join the project***		
	**Pain reduction**	The potential for significant pain relief.	*“Yes*,* it (the pain) got worse after exercising (previous treatment)*,* but it decreased in the evening and then I realized it was better than it was before I started exercising. That’s why I wanted one at home*,* I thought this might be a good thing.” - S3* *“I want to see if there is any relief for the pain.” – S5*
	**Contribute to research**	Opportunity to contribute to research.	*“I’m happy to join and continue to help*,* even if I don’t have a problem*,* it can help others*,* that’s good*,* all for the sake of research.” – S6* *“Well it’s to help move development forward*,* I know how horrible it is to have these phantom pains and if you can help find some way to prevent people from this in the future that would be great.” -S6*
	**Know something new**	Learning about and utilizing new technologies and methods.	*“I think it’s fantastic technique to be able to work and I see for myself that I can move*,* so I think that gave me good feedback*,* that I could do these exercises.” -S6* *“I think it’s kind of cool that you can measure muscle activity in this way and that you can imagine that you’re moving the leg*,* and you get it visually*,* that it’s moving. It’s feedback that motivates you to train*,* I think it’s fantastic that you can work that way.” -S6*
	***To continue train at home***		
	**Pain reduction**	Continued training driving by ongoing benefits in managing pain.	*“I would normally get an attack every three or four days*,* every time a new (weather) front comes through*,* sometime mild*,* sometimes intense but regularly more than once a week and this whole season the last twelve weeks (during at-home training) I think I had three or four attacks. That’s also a massive reduction. My friends have noticed it as well and that’s fantastic.” – S4*
	**New technology brings hope**	Technology provides hope and motivation for sustained engagement.	*“Now I can sleep*,* and I can dream. ”- S4*
**Usability**	**Easy to learn and use**	Time and effort needed to learn and simplicity of the technology to be used (at home).	*“The water is easy (to place on the band) and everything is extremely easy and simple.” -S2* *“For me*,* I just asked my family to help me fix it for the first few times. Later*,* it was me. Because he was often away from home sometimes*,* no one helped me*,* so I stuck it first and put it on.” – S5* *“So I think the design of this system is quite intelligent. For me*,* it’s not particularly difficult*,* but it also has some requirements and some difficulties (in the beginning).” -S5* *“Also it become quick and easy*,* you get used to it (preparing the band). I almost an expert in that now…” -S4*
	**Comfort to use**	Comfortable for regular use	*“No problem at all*,* it’s perfectly fine. (to wear the band)” -S6* *“I was very pleasantly surprised*,* I thought this (the band) will break*,* it will fall apart*,* but it attached great with velcro. It’s really comfortable*,* it doesn’t feel like a problem to have on that band*,* it works as well as anything.” – S6* *“No*,* you hardly feel it when you wear it*,* that piece is as good as it gets.” – S6*
	**System robustness**,** reliability and accuracy**	Hardware robustness, software reliability and the overall accuracy of the information the users received.	*“It (the band) finds the signals*,* that’s guaranteed*,* and you didn’t have to turn it*,* you just put it on.” – S3* *“The program (software) has worked great*,* there has never been a problem starting it*,* there has been nothing.” – S6* *“It (software) has worked fantastically well. There were times when I had to go in and out*,* but otherwise I have never experienced any problems.” – S6* *“Technically the system works brilliantly*,* it’s never disconnected from the laptop to the dongle*,* the battery last really long in the little device itself. It’s nice and clean and feels robust*,* nothing looks likes it wearing out yet. It seems to be in very good condition*,* it’s very good quality.” -S4*
	**Availability**	Accessible for use at any time, any place.	*“I am really busy from Monday to Sunday without any rest. The only rest is that when my children feel asleep*,* I can sit down and take a bath at night*,* and then I can practice my hands and watch TV.” -S5* *“I can tell you for sure that if life was as normal I’d definitively be able to use it once a week*,* no problem because I got a special place for it and then I just pull it out when I need it.” -S4*
	**Compatibility**	Compatible with daily routines, incorporate into regular activities and fit individuals schedule and needs	*“I used to maintain three times*,* but then I reduced it to two times. In the last two weeks or so*,* I basically didn’t practice*,* because I was really too busy.” -S5* *“I made it part of my weekly routine*,* like people going to church on Sundays. I dedicated that time to myself—for my mind and body—taking the weekend to focus without feeling rushed.” -S4*
	**Saving effort and time**	Time and effort saved by the new technology.	*“I told my facilitator*,* if I should do this (the training) at home*,* you have to find the position of the electrodes and I have to draw with a marker and then make a tattoo where they should be so it remains*,* but then the band arrived and it works great… “*-S3 *“Overall experience*,* it’s great*,* it’s thousand times better than all the individual things and I do enjoy not having my hair pulled out of my leg every time I remove them.” – S4*
**Support**	**From the professionals**	Professionals” guidance including personal support, reminders, training plan, etc.	*“I think it’s good (the weekly emails). Very clear guidance*,* written requirements*,* so very clear” -S5* *“I feel very good (about the given support)*,* any questions can be answered in time*,* communication is also very good*,* very timely*,* so I am very satisfied.” -S5* *“I think… the time I spent physically with you and the team… was helpful in the time when we weren’t having any email contact. I think my connection to you as people definitely motivated me more to show up… “– S4*
	**From the family**	Practical assistance during the interventions from the family/next-to-kins	*“For me*,* I just asked my family to help me fix it for the first few times. Later*,* it was me. Because he was often away from home sometimes*,* no one helped me*,* so I stuck it first and put it on.” – S5* *“Because I can’t fix it myself*,* I can’t stick it if I pull it up from there and then put it on.“- S5*
	**Having own community**	Sharing experiences and emotional support with people in the same condition.	*“I think the sense of belonging and I almost feel like if I was in touch with some other amputees who were using the machine like a little group it would make a massive difference.” – S4* *“I’ve been an amputee for nearly twenty years*,* next year will be twenty years and I can tell you something*,* what’s most common is the sense of isolation because nobody really knows what you go through*,* nobody cares because they don’t know that it’s relentless. If I have to tell people every time I have a challenge or phantom pains*,* they would just think I’m making it up. What’s the matter with this guy? If you got other amputees*,* I think it would make a massive difference to just be part of something bigger…I think that the psychological connection is probably as important and the emotional connection is probably as important as the machine and the work itself*,* if I have to be honest.” – S4*
