# Supplementary material for: Towards the implementation of home-based phantom limb pain training facilitated by a textile-electrode system: lessons learned from a pilot study
Source: J Neuroeng Rehabil. 2026 Mar 11;23:119. doi: 10.1186/s12984-026-01923-w (PMC13063768; doi:10.1186/s12984-026-01923-w)
Supplement: Supplementary file 1 — Supplementary Material 1 [file 12984_2026_1923_MOESM1_ESM.docx]

**Semi-Structured Interview Guide**

**Introduction**

Thank you for agreeing to participate in this interview. We are interviewing you to better understand what patients think about the PLP treatment and the system in general but also specifically the textrode-band and how these may be improved to better facilitate the home-based treatment for people with Phantom Limb Pain. So there are no right or wrong answers to any of our questions, we are interested to learn more about your own experiences from using the PLP treatment at home.

Participation in this study is voluntary and your decision to participate, or not participate, will not affect your further treatment. We anticipate the interview to go on for approximately one hour depending on how much information you would like to share. With your permission, I would like to audio record the interview because I don’t want to miss any of your comments. All responses will be kept confidential. This means that your interview responses will be transcribed, and the transcriptions will only be shared within the research team members without any reference to your name in order to ensure that any information that will be included in the report does not identify you as the respondent. You may decline to answer any question or stop the interview at any time without being required to state any reason.

So, before we proceed, do you have any questions or is it something you want to be explained in more detail?

Are there any questions about what I have just explained?

May I turn on the digital recorder?

*Note 1: this guide only represents the main themes to be discussed with the participant. Prompts examples are given for each question, other various prompts including non-leading and general prompts can also be used, such as ‘Can you please tell me a bit more about that?’ and ‘what does that look like for you?’*.

*Note 2:* Questions with ‘*’ will only be answered by the patients who have previous experience in using the Neuromotus system.

Note 3: Questions with ‘**’ will be asked for the 2^nd^ interview only.

**Establishing Rapport**

(Before the interview begins, some icebreaking questions)

How are you today? / How was your weekend? /

**Overall impression**
**Prompts:** First, can you just tell about your general thoughts/experiences about/from using the PLP treatment (the last three months). What have been positive? Are there any drawbacks/disappointments? Do you see any long-term changes in your use of the PLP system during the last three months compared to previous use? (Any thoughts about the introduction/guidance to use the system?) What is your primary motivation to use the system (taking part in the study? Pain relief? Health effects/“Feels good”?)

1. ***Previous experiences in using the Neuromotus system for PLP treatment**

**Prompts**: Please tell us a bit about your previous experience using the Neuromotus system.

For example, how long have you been using the Neuromotus system? Where do you usually receive the treatment? In clinic or at home? How often do you receive the treatment? Can you remember what was your primary motivation to use the system at “that time”?

**Prompts:** was it a team that provided treatment for you? Was it the quality of care you received? Was it the ability to access care?

**Prompts**: For each training session, how long time do you usually spend on preparation and the actual treatment?

**Prompts**: Was there anything you particularly liked about the treatment? Was there anything you didn’t like about it? Was it up to your expectations?

1. **Patient experiences of the current treatment/ intervention**

**Prompts**: In thinking about the treatment you received in the previous 3 months (i.e. 1^st^ or 2^nd^ period), how would you describe your overall experience? For example, the usability, treatment length and how often you use the system, the assistance/guidance from the team.

** **Prompts**: was there anything you didn’t like about the treatment when it is totally controlled by yourself? Any changes in comparison to “previous”/1st period.

** **Prompts**: How would you describe the 2nd period compared with the 1st period?

**Prompts**: Which aspects would you like to be improved in further developments of the treatment, how would you like to design or improve it? Any thoughts about where, when and how? (Home environment/clinic? Or other place, the treatment length and how many times a week, with or without an assistant. Regular based or do it when needed/free, according to your own will?

1. **Patient experiences of the textrode band**

**Prompts**: In thinking about the overall experience with the textrode-band during the last 3/6 months (i.e. 1^st^ and/or 2^nd^ period), how would you describe your overall experience? What was good? Any drawbacks/disappointments? please describe as detailed as possible!

**Prompts**: let’s talk more in details. Concerning the textrode-band, how would you describe the overall ease of use? Any concerns? The preparation for the band, for example, to wet the electrodes and to connect the cables. How often do you re-connect the cables? How about durability? (Velcro, band elasticity and overall functionality)

**Prompts**: What’s your opinion on wearing the band? And the overall comfort? Did you feel comfortable using it?

* **Prompts**: Compared with your previous gel electrodes experience, how would you describe your experience using the textrode band? Do you prefer one over the other? And why?

**Prompts**: For you, what are the advantages and disadvantages of textrode-band?

**Prompts**: What aspects of the textrode band would you say should be addressed in further developments? How would you like to improve it?

1. **Any changes in patient-reported health status/ outcomes/behaviour related to the -treatment**

How would you describe your health since receiving the treatment? Any changes during the last three months?

**Prompts**: For example, has your health improved, declined or remained the same?

**Prompts**: What are the reasons you think your health has improved, declined or remained the same?

Thinking back to how you handled your care before, can you describe any situations where you would take different steps in managing your care now (e.g. going to the clinic once per month)?

**Prompts**: For example, if you were to suddenly feel unwell, who would you call?

That was the last part of the interview. Do you have any further comments you would like to add? Are there any aspects of the treatment and/or the textrode band that we haven’t covered? Is there any question you thought we would ask but didn’t?

Thank you very much for your time and the information you shared today!
